# Ecotoxicological Effects of Polystyrene Particle Mix (20, 200, and 430 µm) on *Cyprinus carpio*

**DOI:** 10.3390/toxics13040246

**Published:** 2025-03-26

**Authors:** Ştefania Gheorghe, Anca-Maria Pătraşcu, Catălina Stoica, Mihaela Balas, Laura Feodorov

**Affiliations:** 1 Control Pollution Department, National Research and Development Institute for Industrial Ecology ECOIND, 57-73, Drumul Podu Dambovitei Str., 060652 Bucharest, Romania; anca.harabagiu@ecoind.ro (A.-M.P.); catalina.stoica@incdecoind.ro (C.S.); laura.feodorov@ecoind.ro (L.F.); 2 Faculty of Biotechnical Systems Engineering, National University of Science and Technology Polyethnic, 313 Splaiul Independentei, 060042 Bucharest, Romania; 3 Department of Biochemistry and Molecular Biology, Faculty of Biology, University of Bucharest, 91-95 Splaiul Independentei, 050095 Bucharest, Romania; 4 Faculty of Biotechnology, University of Agronomic Sciences and Veterinary Medicine of Bucharest, 59 Marasti Blvd, District 1, 011464 Bucharest, Romania

**Keywords:** *Cyprinus carpio*, microplastics, polystyrene, enzymes, oxidative stress

## Abstract

Global consumption led to increased and persistent plastic pollution in aquatic environments, affecting aquatic biota. Polystyrene (PS) is a synthetic polymer and one of the most widely used plastics. This study aims to investigate the acute and chronic effects of PS microplastics on *Cyprinus carpio* using an adapted OECD methodology. For the acute test, PS was tested in different particle sizes (20, 200, and 430 µm), each at concentrations of 0, 1, 10, and 100 mg PS/L. Mortality and clinical signs were monitored after 96 h of exposure. No acute effects were recorded. In the chronic test, a mix of PS particles of different sizes (20, 200, and 430 µm) at a total concentration of 1.2 mg PS/L was used for a 75-day fish exposure. Mortality, biometric parameters, physiological indices, and antioxidant enzyme activities, including catalase (CAT), glutathione reductase (GRed), glutathione S-transferase (GST), 7-ethoxyresorufin-O-deethylase (EROD), lipid peroxidation (MDA), hepatic enzymes (alanine aminotransferase—ALT and aspartate aminotransferase—AST), vitellogenin (VTG), and acetylcholinesterase (ACh), were assessed. Fish exposed to the PS mix exhibited a 40% change in hepatosomatic indices after 75 days. Additionally, the PS mix induced oxidative stress in fish organs. CAT activity increased fourfold in the intestine, GRed activity increased thirtyfold in the gonads, and GST activity doubled in the brain. GRed activity also increased in the gills but was not statistically significant compared to the control. Lipid peroxidation was observed in the kidney (twofold increase) and was also detected in the gills and intestine; however, these changes were not statistically significant. EROD activity increased by 15% compared to the control group, indicating an amplification of stress enzyme expression. The activity of hepatic enzymes ALT and AST increased nine to tenfold compared to the control. VTG activity increased by 47%, and ACh activity showed more than 80% inhibition in the brain and muscle. Furthermore, an overall amplification of protein expression in the intestine and liver was observed compared to the control group. Our study revealed the incidence and severity of PS microplastic effects on freshwater fish and emphasized the urgent need for prevention, monitoring, and mitigation measures to combat microplastic pollution.

## 1. Introduction

The increasing presence of plastic waste in nature raised significant concerns among researchers due to its detrimental impact on ecosystems. According to Plastics Europe, global plastic production increased drastically over recent decades, reaching approximately 400 megatons in 2022 [1,2]. Despite this, data from the Organization for Economic Co-operation and Development (OECD) indicate that plastic consumption declined by 2.5% in 2020 compared to 2019 but increased again in 2021. Alarmingly, only 17% of plastic waste is currently recycled [3]. Each year, an estimated 8 million tons of plastic enter the world’s oceans [4], largely originating from packaging waste, improper waste management, and major consumer goods producers [3].

Microplastics, defined as plastic fragments smaller than 5 mm, originate primarily from the degradation of larger plastic debris over time [5]. They exist in various forms, including spheres, fibers, and irregular fragments [6], and exhibit a wide range of colors [7]. These particles represent an intermediate stage between macroplastic pollution and nano-plastics, which pose even greater environmental risks. The widespread contamination of aquatic ecosystems with microplastics has been documented in oceans [8,9], seas [10,11], sediment [12,13], fresh water [7,14,15], and wastewater [16]. This contamination poses major ecological dangers as well as hazardous impacts on organisms [2], microplastics being identified in fish and marine invertebrates [10,12].

The most common microplastics identified are polyester, polyethylene, polyethylene terephthalate, polyamide, polypropylene, polystyrene, and polyurethane [17]. For example, in Europe, the types of microplastics found in aquatic environments include polypropylene (16.28%), polyethylene (13.95%), polystyrene (6.98%), and other plastics (>50%), with their size ranging from <25 μm to 100,000 μm [3]. The abundance of microplastics is expressed in various units in the literature, such as the number of particles/items per liter (L), cubic meter (m^3^), square meter (m^2^), kilogram (kg), or milligram (mg). Depending on the source of the sample, microplastics have been reported in significant concentrations. For instance, in wastewater influents and effluents, 10^−5^ to 10^10^ particles per m^3^; in surface water, 0–10^3^ particles per m^3^ or 0.008 mg/L to 0.039 mg/L; and in sediment, 2 × 10^3^–1.6 × 10^4^ particles per kg. The concentrations vary depending on the pollution levels and environmental conditions [4,18].

The presence of polyethylene, polypropylene, and polystyrene has also been registered in the surface water of Romania, specifically in the Danube River and Somesul Mic River [19,20]. In the last decade, the scientific community paid increased attention to the presence of microplastics in the environment due to their toxicity to humans, as well as aquatic and terrestrial organisms [1,16]. Most environmental studies focused on the impact of microplastic on marine organisms [9,12,13], followed by freshwater organisms [3,15]. Because microplastic particles are tiny and mimic natural food sources, fish can easily ingest them [21]. This ingestion can affect fish not only physically—causing gastrointestinal tract obstruction and mechanical damage due to accumulation—but also behaviorally, altering swimming patterns, feeding habitats, reproductive abilities, and development processes. Additionally, microplastics can influence fish metabolically by enhancing oxidative stress responses through increased generation of reactive oxygen species (ROS) and impairing immune responses due to their ability to absorb additional environmental contaminants that bioaccumulate [17,22,23,24,25,26].

Polystyrene (PS) is produced through the polymerization of styrene monomers and is widely used due to its low cost, durability, and diversity of uses. It is utilized in various applications, including food packaging, toys, compact discs (CDs), construction materials, and more [27]. According to Statista 2024, global polystyrene production has shown slight growth compared to 2022 [28]. However, the main issue with polystyrene is in its stability. PS biodegrades in natural environments, but the process is so slow that it is generally considered non-biodegradable [29,30]. Hollerova et al. (2023) suggest that PS microplastics can affect the health of freshwater fish (*Oncorhynchus mykiss*), causing severe damages in the liver and gills as well as altering fish behavior [31]. The accumulation of PS in fish tissues (e.g., *Oreochromis niloticus*, *Oryzias latipes*, and *Oryzias javanicus*) can lead to metabolic disturbances and oxidative damage in target organs such as the intestine, gills, liver, or brain [32]. A comprehensive review of the hazards associated with polystyrene (PS) exposure in aquatic organisms indicates that PS particles—whether microplastic or nanoplastic—can elicit a range of toxicological effects. At the individual level, PS exposure can disrupt normal body development and reproductive functions [33]. At the cellular level, it induces histopathological alterations, DNA damage, and apoptosis. At the molecular level, PS exposure has been shown to cause oxidative stress, neurotoxicity, and genotoxicity [34].

Microplastics have been detected in various fish species, particularly in *Cyprinus carpio* in their natural habitats. Laboratory identification analyses revealed the ingested particles polyethylene (PE), polypropylene (PP), and polystyrene (PS) [35]. For example, a study on *Cyprinus carpio* from the Thames River, Ontario, found microplastics in 31% of common carp, with higher concentrations in urban areas compared to rural sites [36]. In contrast, in laboratory studies, PS accounted for 41.95% of the plastics used in the experiments, due to an easy availability and a similar density with water [37]. A recent study on the Danube River basin showed the accumulation of PS in the muscle of *Mytilus galloprovincialis*, which can pose a real risk to human health through its utilization as food [38]. Another study identified PS fragments in commercial fish meal and cultured carp, raising concerns about contamination in aquaculture systems [35].

Recent review studies highlight critical knowledge gaps in microplastic sampling protocols, contamination control, hydrogeological properties, interactions between microplastics and biota, and the long-term ecological and health impacts of microplastic contamination [39,40,41]. Further studies are required to investigate the presence and effects of microplastics in fish organs and edible tissues to better understand their translocation in aquatic organisms and potential uptake by humans [42]. Studies reported that significant percentages of fish sold in markets are contaminated with microplastics, raising questions about food safety and long-term health implications [40].

Our study revealed the acute (96 h) and chronic (75-day) effects of polystyrene (PS) microplastics exposure on *Cyprinus carpio* (common carp), utilizing spherical PS particles of three distinct sizes (20, 200, and 430 µm). The research aims to highlight the toxicological impacts at the individual, cellular, and molecular levels. The collected data include information on the following: biometric parameters; physiological indices; antioxidant enzyme activities (catalase [CAT], glutathione reductase [GRed], glutathione S-transferase [GST]), 7-ethoxyresorufin-O-deethylase (EROD) activity, and lipid peroxidation); hepatic dysfunction (Alanine aminotransferase [ALT] and aspartate aminotransferase [AST] activities); neurological and reproductive effects (vitellogenin [VTG] and acetylcholinesterase [Ach]); and protein expression. The study will provide scientific insights into how PS exposure affects an aquaculture species and help understand microplastic pollution in freshwater ecosystems.

## 2. Materials and Methods

### 2.1. Chemicals

The experiment contaminant was polystyrene (PS) microspheres (CAS 9003-53-6) with the following properties: colorless, density 1.05 g/mL, sizes of 20, 200, and 430 µm. Particles were suspended in 10% aqueous solutions (Sigma-Aldrich, Saint Louis, MO, USA).

The spherical form of PS was selected for this study based on the following: (1) Its prevalence as a common microplastic type in aquatic environments [43]. PS particles have been identified in Someșul Mic River (Romania), where spherical PS particles ≤ 500 µm were detected at all sampling sites [44]; (2) high bioavailability via ingestion or inhalation by aquatic organisms and potential for acute toxic effects [45]; (3) commercial availability; and (4) ease of visualization under microscopy using specific staining techniques.

Other used reagents included the following: hydrogen peroxide (H_2_O_2_ 30%) necessary for polystyrene separation from test samples; Nile Red for fluorescence microscopy; and biochemical and electrophoresis reagents (>98% purity) provided by Sigma-Aldrich (Saint Louis, MO, USA) and BioRad (Hercules, CA, USA).

### 2.2. Fish

*Cyprinus carpio* (common carp), a freshwater fish prevalent in European surface waters, is a widely used model organism for environmental studies, particularly in assessing the impacts of pollutants on freshwater ecosystems [46], and was used in our research. Healthy specimens were obtained from an unpolluted fish farm of the Fisheries Research and Development Station Nucet, Romania, and acclimated to laboratory conditions at the National Research and Development Institute for Industrial Ecology (ECOIND) in Bucharest, Romania.

The in vivo studies adhered to the Guide for the Use and Care of Laboratory Animals and the OECD recommendations to minimize animal suffering and the number of animals used [47]. This research received authorization and close supervision from the ECOIND Commission of Ethics and Professional Deontology (Internal Procedure no. 4336/28.03.2022). The acclimatization conditions of fish consisted of the following: 800-L aquariums with water supply systems, water filtration systems, and lighting systems; acclimatization water was chlorine tap water with dissolved oxygen 6.23 ± 1.5 mgO_2_/L (using air pumps with flow meters), 7.32 ± 1.31 pH unities, temperature 20 ± 2 °C, water hardness 197 ± 21 mg CaCO_3_/L, free chlorine < 0.03 mg/L, nitrite 0.03 ± 0.001 mg/L, nitrate < 0.5 mg/L, and un-ionized ammonia < 0.001 mg/L; the fish were fed daily with 1% of the lot’s weight/aquarium; the acclimatization period was 2 weeks from the fish reception in the laboratory. Only healthy specimens without visible lesions and with normal mobility were used in the next laboratory testing.

Given the controlled conditions of the fish farm and the use of a parallel control test, the risk of microplastic contamination prior to the experiments was likely minimized. However, it is worth noting that complete elimination of pre-existing microplastic exposure is challenging, as these particles are ubiquitous in aquatic environments [35,48].

### 2.3. Acute Toxicity Test 

The acute toxicity test of PS particles was conducted using adapted test conditions-based OECD 203 in a static system [49]. The PS was tested in the different particle sizes of 20, 200, and 430 µm, each at concentrations of 0, 1, 10, and 100 mg PS/L. The initial estimated density of the particles in the exposure tests was as follows: 2.5 × 10^5^ to 2.5 × 10^7^ (number of particles for the PS size 20 µm per L); 2.5 × 10^2^ to 2.5 × 10^4^ (number of particles 200 µm per L); and 25 to 2.5 × 10^3^ (number of particles 430 µm per L).

Testing groups included five one-year-old *Cyprinus carpio* individuals per each test or control group, with a weight of 32.64 ± 5.5 g/individual and length of 11.60 ± 3 cm/individual. The PS particles were added to the test aquariums in parallel with the food (1% of the lot’s weight) only once at the start of the test. Experimental conditions consisted of a static system with an exposure time of 96 h, a temperature range of 18–25 °C, and a light cycle of 12–16 h/day. Control groups were maintained with dilution water (potable water free of chlorine). The solution volume was set at 10 L for both test and control groups. The endpoints of the experiment were the mortality and clinical signs of sublethal effects.

The polymer particles from the test solutions were highlighted microscopically using the oxidation with hydrogen peroxide (30%) followed by Nile Red staining (solution of 10 µg/mL in acetone), filtration through a 0.45 µm filter, and two consecutive rinses with distilled water. To prevent additional contamination with microplastics, the test vessels were permanently covered. The handling of the samples was carried out in a chemical fume and the samples were kept in covered glassware.

### 2.4. Chronic Toxicity Test

The chronic toxicity of PS particles was assessed using an adapted OECD 305 methodology [50] and was carried out in a single replicate. In this case, a PS mix of three different particle sizes of 20 µm, 200 µm, and 430 µm initially stained with Red Nile, was used for intoxication. The total concentration of PS mix was 1.2 mg /L (approximately 0.4 mg/L for each size). The initial estimated density of the particles in the exposure tests was as follows: 1 × 10^5^ (number of particles PS 20 µm per L); 1 × 10^2^ (number of particles PS 200 µm per L); and 10 (number of particles PS 430 µm per L).

The PS mixes were added every 48 h in parallel with the food. The test and control group, each included 10 fish older than 2 years, with a weight of 68.63 ± 10 g/individual and average length of 15.9 ± 1 cm/individual. The fish were fed daily with commercial food at a rate of 1% of the lot’s weight.

The experimental conditions consisted of a semi-static system with the replacement of 80% of the test solutions every 48 h, an exposure duration of 75 days, a temperature range of 18–25 °C, a light cycle 12–16 h/day, and control with dilution water (potable water free of chlorine). The total volume of test solution was set at 80 L for each test or control group.

The endpoints of the experiment included mortality, sublethal clinical sigs, biometric indicators, physiological indices, and hepato/gonadosomatic indices. After 75 days of exposure to PS, three fish from both test and control fish groups were euthanized through cervical transection on ice. To evaluate the effect of PS on the enzymatic systems, the fish organs (liver, gills, kidneys, intestines, brain, gonad, and muscles) were collected individually.

In both tests (acute and chronic), the quality of dilution water used in the preparation of the test solutions and controls, as well as the test solutions themselves, were periodically analyzed for physical and chemical parameters according to OECD guidelines.

### 2.5. Investigation of PS Microplastics in Test Solutions and Body Accumulation

Using glass containers and stainless-steel laboratory utensils, testing solution and control samples, as well as intestinal content samples, were collected to investigate the presence of particles in the test media and in the fish body after the exposure period. A volume of 500 mL of testing solution/control solution was evaporated for 24 h at 90 °C and treated with 30% hydrogen peroxide (at temperatures above 75 °C) for organic matter digestion. Approximately 0.1 g of intestinal content samples were directly treated with 20 mL of 30% hydrogen peroxide. The digested samples were filtered through a 0.45 µm filter and analyzed under a microscope. Based on the fluorescence obtained after the initial PS particle staining with Nile Red, their presence was investigated using a Leica DMi8 inverted microscope (Leica Microsystems, Wetzlar, Germany, software LAS V4.7).

### 2.6. Biometric Data, Physiological Indices, Hepato/Gonadosomatic Index

In the chronic experimental setup, the individual weight, length, and height of the fish were measured at 0 h and after 75 days of exposure to the PS mix. Based on the biometric data of the tested fish, the following were calculated [51,52]: the instantaneous growth coefficient—G (Equation (1)); the instantaneous mortality coefficient—Z (Equation (2)); the efficiency of food use for production—K (Equation (3)); biomass—B (Equation (4)); fish production—P (Equation (3)); and food consumption—C. In addition, the liver and the gonads of each fish were weighed to calculate the hepatosomatic index (HSI = liver weight/body weight × 100) [53] and gonadosomatic index (GSI = gonad weight/body weight × 100) [54].

(1) $$G=\frac{\log_{e}\overset{-}{W_{f}}-\log_{e}\overset{-}{W_{i}}}{\Delta T}$$

where

- W_i_, W_f_—initial and final average weight of the fish;
- ΔT—experimental period (days);

(2) $$Z=\frac{\log_{e}N_{i}-\log_{e}N_{f}}{\Delta T}$$

where

- N_i_, N_f_—initial and final number of specimens (fish);
- ΔT—experimental period (days);

(3) $$K1\%=\frac{P}{C}\times 100\;$$

where

- C—food consumption;
- P—fish production = B × G;
- G—instantaneous growth coefficient;
- B—biomass average, is calculated from the relations;
- Bi—initial biomass = N_i_ × W_i;_

(4) $$\bar{B}=\frac{B_{i}\left(e^{G-Z}-1\right)}{G-Z}$$

where

- B—biomass average;
- Bi—initial biomass = N_i_ × W_i;_
- G—instantaneous growth coefficient;
- Z—instantaneous mortality coefficient.

### 2.7. Biochemical Analyses

#### 2.7.1. Tissue Homogenate Preparation

Organ samples were mechanically homogenized using an MT-13K-L mini handheld homogenizer (Miulab, Hangzhou, China) in a 1.5 mL Eppendorf tube with 500 μL of lysis buffer containing Tris-HCl (150 mM) with 5 mM EDTA (pH = 7.4, ratio 1:10 *m*/*v*) and 2% sodium dodecyl sulfate (SDS). Subsequently, an additional 500 μL of lysis buffer, including 10 µL of 2 mM sodium fluoride phenylmethylsulphonyl fluoride (PMSF, a serine protease inhibitor), was added to achieve a final volume of 1 mL. The samples were vortexed for 2 min using an FVL-2400N Combi-Spin vortex (BIOSAN, Riga, Latvia). After tissue homogenization and cell lysis, the samples were incubated for 1 h at 4 °C and centrifuged for 10 min at 12,298 RCF and 4 °C. The supernatant (500 µL each /aliquot) was refrigerated at −80 °C for subsequent biochemical analyses.

The protein concentration in the tissue homogenates was measured using the Lowry method [55] by reading the absorbance of the final products at 660 nm. A standard curve was prepared using a 10% bovine serum albumin solution with a concentration range of 0–90 µg/mL and an *r*^2^ value of 0.99007.

#### 2.7.2. Enzymatic Activity Measurement and Lipid Peroxidation

The enzymatic activity of catalase (CAT), glutathione S-transferase (GST), and glutathione reductase—(GRed) were analyzed spectrometrically. Optical densities of the reaction products were measured using a Shimadzu UV 1900 (Duisburg, Germany) and a ClarioStar Multi-Plate Reader (BMG Labtech, Ortenberg, Germany).

CAT activity was measured using the Aebi method by monitoring the reduction in H_2_O_2_ concentration at 240 nm. One unit of CAT activity is defined as the decomposition of one micromole (µmol) of H_2_O_2_ per minute per milligram of protein [56].

GST activity was assessed following the Sigma-Aldrich kit protocol (MAK453, Sigma-Aldrich, Saint Louis, MO, USA) at 340 nm by measuring the rate of conjugation of 1-chloro-2,4-dinitrobenzene (CDNB) with reduced glutathione (GSH). One unit of GST activity corresponds to the formation of one µmol of conjugated product per minute. An extinction coefficient of 9.6 mM^−1^ cm^−1^ for CDNB was used in the calculations.

GRed activity was measured using the method described by Goldberg and Spooner, involving a mix of 0.1 M phosphate buffer (pH 7.4), 0.66 mM oxidized glutathione (GSSG), and 0.1 mM nicotinamide adenine dinucleotide phosphate—reduced form (NADPH). One unit of GRed activity was defined as the consumption of one µmol of NADPH per minute [57].

The hepatic enzymes alanine aminotransferase (ALT) and aspartate aminotransferase (AST) were spectrometrically analyzed at 340 nm using Randox kits (IFCC Manual, AL 1205 for ALT and AL 1204 for AST, Randox Laboratories Ltd., County Antrim, UK). ALT activity was determined based on the reaction between α-oxoglutarate and L-alanine, producing L-glutamate and pyruvate through the catalytic action of ALT. AST activity was measured based on the reaction between 2-oxoglutarate and L-aspartate, producing L-glutamate and oxaloacetate through AST catalysis.

The acetylcholinesterase (ACh) activity was evaluated by measuring the colorimetric product at 412 nm resulting from its reaction with 5,5′-dithiobis (2-nitrobenzoic acid). The analysis was performed at 20–25 °C using the Sigma-Aldrich kit MAK119 (Saint Louis, MO, USA).

EROD (7-ethoxy-resorufin-O-deethylase) and vitellogenin (VTG) activities were analyzed using ELISA kits (MBS1601687 and MBS1603087 from MyBioSource Inc., San Diego, CA, USA). These kits rely on antibody–antigen interactions combined with a horseradish peroxidase (HRP) colorimetric detection system to identify VTG/EROD antigens in samples. Absorbance readings were performed at 450 nm. Standard curves were used for calculations: EROD range 1.5–24 ng/mL, *r*^2^ = 0.9753 and VTG range 15–240 µg/mL, *r*^2^ = 0.9753.

All enzyme activities were normalized to protein concentrations and expressed in terms of activity units per milligram of protein (U/mg protein).

Lipid peroxidation was assessed by measuring malondialdehyde (MDA) levels based on its reaction with thiobarbituric acid (TBA), forming a colorimetric product proportional to MDA presence in samples. Absorbance readings were performed at 532 nm using the Sigma-Aldrich MAK085 kit (Saint Louis, MO, USA) according to manufacturer instructions. A standard curve ranging from 0 to 100 nmol/mL (*r*^2^ = 0.9358) was used to estimate MDA levels, expressed as nmoles of MDA/mg protein.

### 2.8. Assessment of Protein Expression Profile

Protein profiles were performed for the intestine, gills, and liver, as these tissues are specific to the exposure routes. Proteins were extracted using the same procedure applied for tissue homogenate preparation and separated by electrophoresis on 10% polyacrylamide gels under denaturing conditions (SDS-PAGE). Thermal denaturation of the samples was carried out at 95 °C for 15 min at 600 rpm using an Eppendorf Thermomixer (Madrid, Spain). A total of 50 µg of protein from each sample was loaded onto the gel. Alongside the samples, a standard molecular mass marker, Precision Plus Protein Standards Dual Color (BioRad, Hercules, CA, USA), was also loaded.

Protein migration was performed in the presence of Tris/Glycine/SDS 1× electrophoresis buffer (BioRad, Hercules, CA, USA) at a voltage of 100–150 V. After separation, the gel was fixed with a solution containing 40% ethanol and 10% acetic acid for 15 min, washed, and stained with Coomassie blue at room temperature overnight with gentle agitation using an Ika KS 130 basic shaker (Staufen, Germany). The gels were de-stained in deionized water for one hour with three washes until protein bands became visible.

Protein bands were visualized using a Syngene™ Transilluminator for G:Box and analysed with GeneSys software version 1.5.2.0 (Thermo Fisher Scientific, Waltham, MA, USA). Quantification of protein bands in each sample was performed using GelQuant.NET software, version 1.8.2.

### 2.9. Statistical Analysis

The results of the monitored physical and chemical parameters and biometric data used in toxicity tests are presented as mean ± standard deviation (SD) for n = 4 (in acute toxicity test) and n = 45 (in chronic toxicity test) determinations or n = 10 fish specimens and calculated using Microsoft Excel 2016. The results of HIS, GSI, and biochemical analyses are presented as the mean ± standard deviation (SD) of n *=* 3 fish specimens. Statistical analysis was performed using GraphPad Prism (version 8, GraphPad Software, La Jolla, CA, USA). Multiple *t*-tests (one per organ) were performed on the obtained data. Statistical significance between the control and PS-treated group was determined using the Holm–Sidak method, with alpha = 0.05. The levels of significance were defined as follows: *p* ≤ 0.05 indicates a statistically significant difference (5% probability of the result occurring by chance), ** *p* ≤ 0.01 indicates a highly significant difference (1% probability of error), and *** *p* ≤ 0.001 indicates an extremely significant difference (0.1% probability of error. The Shapiro–Wilk test for each group (PS-treated groups and untreated groups) within each organ was conducted to assess normality.

## 3. Results and Discussions

### 3.1. Acute Toxicity Effects

The main objective of the acute toxicity test was to observe whether lethal effects occur after exposure of fish to PS for a short period of time (96 h).

The dilution water (dechlorinated tap water) was analyzed from a physico-chemical perspective at the beginning of the tests. It was found that the analyzed indicators correspond qualitatively to the OECD guidelines: pH 7.86 ± 0.50 unities; total hardness 121 ± 9.30 mg/L CaCO_3_; chemical oxygen demand (COD) 10 ± 2.15 mgO_2_/L; suspended solids 4.2 ± 0.41 mg/L; filterable residue 168 ± 12.30 mg/L; ammonia < 0.02 mg/L; residual free chlorine < 0.03 mg/L; dissolved oxygen 7.34 ± 1.24 mgO_2_/L; nitrates 3.90 ± 0.12 mg/L; and conductivity 324 ± 12.1 µS/cm.

The parameters essential for fish survival were monitored daily during the exposure period. The results are presented in Table S1—Supplementary Material. The dissolved oxygen concentration was mentained at ≥6 mgO_2_/L, the pH was 7.33 to 7.76 pH units, and temperature was 21 °C, with COD values between 35 and 69 mgO_2_/L. Acute toxicity experiments with juvenile *C. carpio* showed that PS does not cause acute harmful effects at concentrations up to 100 mg/L after 96 h. No clinical signs, according to OECD 203 (such as loss of equilibrium, anormal swimming behavior, anormal respiration, anormal skin pigmentation, or other clinical signs), were observed in the exposure period and after. No mortality was observed after exposure to 100 mg/L PS microplastics in sizes of 20 to 430 µm after 96 h. The LC50 value could not be determined accurately, as no mortality was observed at any of the tested concentrations; therefore, it can be concluded that the LC_50_ is higher than the maximum tested concentration (100 mg/L) for each size of the tested PS spheres. According to literature, the exposure to PS microplastics result in accumulation in aquatic organisms but without affecting mortality in the short-term toxicity tests [58,59].

The PS solution has a density >1.05 g/mL, which leads to particle deposition on the bottom of the test vessels. Maintaining the particles in suspension within the test system was achieved with the help of aeration devices (consisting of an air pump, flowmeter, hoses, and pumice stones for uniform distribution of air in water). The aerations entrain the microplastic particles in the test system and oxidize the organic matter. The organic loading comes from food administration together with the specific amount of microplastics. The average density of suspended solids in the control group was measured at 7.6, 41, and 78 mg/L, and in the test, solutions containing PS, it was measured at 25.60, 44.50, and 78.33 mg/L, depending on the exposure time (1 day, 2 days, and 4 days).

### 3.2. Investigation of the Presence of PS Microplastic in Test Media and Fish Body

**Figure 1:**
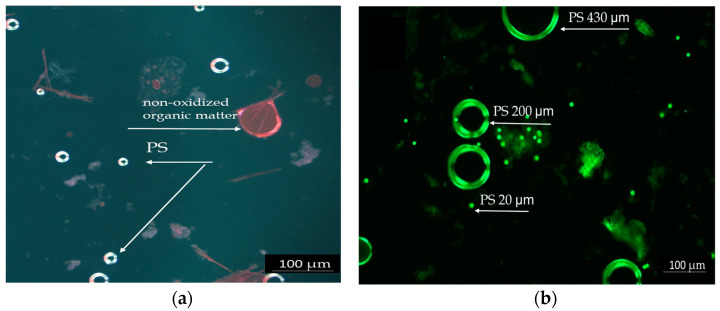
Polystyrene particles highlighted by Red Nile staining: (**a**) PS 20 µm in the intestinal contents of acutely exposed fish, 20×. (**b**) PS particles different dimensions (20, 200, and 430 µm) in the chronic test solutions, 10×, scale 100 µm.

The intestinal content of the fish was investigated microscopically to highlight the ingestion of PS particles. Figure 1 shows PS particles highlighted by Nile Red staining. PS particles measuring 20 µm, separated after the oxidation of intestinal contents with 30% hydrogen peroxide, are shown in the bright field in Figure 1a. The image displays small PS particles measuring 20 µm, which are visible in the intestinal contents of fish that were exposed to PS for a short duration. The particles appear as bright spots due to Nile Red staining, which enhances their visibility under the microscope.

The PS mix (20, 200, and 430 µm) from the test chronic solution is presented in Figure 1b. Due to its spherical shape, PS was easier to observe. The particles appear as bright spheres in different sizes against a dark background. The particles are shown at a lower magnification, allowing for an overview of their distribution and size differences. The Nile Red staining proved to be efficient and reliable due to the spherical form of PS, which enhances dye adhesion and fluorescence detection. The PS particles were more prominently highlighted in acute tests because of the higher concentrations tested compared to chronic tests.

### 3.3. Chronic Toxicity Test Cyprinus carpio

The chronic exposure of fish considered exposure route, ingestion, and inhalation of PS particles to simulate real conditions. The presence of PS in the test system was highlighted by microscopy in the fluorescence field using 10× magnification (Figure 1b).

During the chronic test, no significant changes were observed in the monitored physical and chemical parameters of PS test solution (Table S2—Supplementary Material). The analyzed indicators met the optimal test conditions recommended by OECD. The dissolved oxygen concentration was mentained at ≥6 mgO_2_/L, the pH ranged from 7.42 to 7.93 pH units, the temperature was between 21 and 22 °C, and conductivity ranged from 228 to 273 µS/cm. Under these conditions, the toxicity effects were not influenced by changes in the survival conditions, suggesting that the observed outcomes were primarily due to PS exposure rather than environmental stressors.

The COD during the chronic tests ranged from 10.74 to 31.40 mgO_2_/L in the control and from 10.00 to 83.30 mgO_2_/L in the PS mix test group. Suspended solids were measured at 3–6 mg/L in the control group and 5–39 mg/L in the PS mix test group. Changing the test solutions every 48h helped to maintain a low concentration of organic matter, which is critical for preventing confounding factors that could affect fish health.

The chemical analyses were performed before and after solutions changing, ensuring accurate monitoring of water quality throughout the experiment. Additionally, aeration, periodic manual entrainment of the particles within the solution, and the active swimming of the fish ensured good particles dispersion and better bioavailability.

At the end of the chronic experimental setup, three homogenous specimens from each group were sacrificed for biological sample collection (liver, gill and intestine, gonad, kidney, brain, and muscle). The organ samples collected for biochemical analysis were processed to obtain tissue homogenates and preserved at −80 °C.

### 3.4. Biometric Data

From the analysis of the biometric data after 75 days of exposure to PS (Table 1), it was observed that the fish groups used in the experiment were homogeneous, registering similar weights both in the tests and in the control groups. However, a slight decrease can be observed in the experiments conducted with the PS mix. At the end of the test, the group weight decreases by 6% for the PS-treated group compared to the initial weight at 0 h. This decrease is insignificant, but may be attributed to the presence of PS contamination. Generally, polymer particles exhibit bioavailability primarily through ingestion with food, which can lead to a state of satiety that may result in inefficient food utilization and, consequently, a decrease in body weight. The control group did not lose weight after 75 days, maintaining their initial weight. By the end of the experiment, the length and height of the organisms remained the same as those in the control group and showed no difference from the measurements taken at the start of the study (0 h).

### 3.5. Physiological Indices

**Figure 2:**
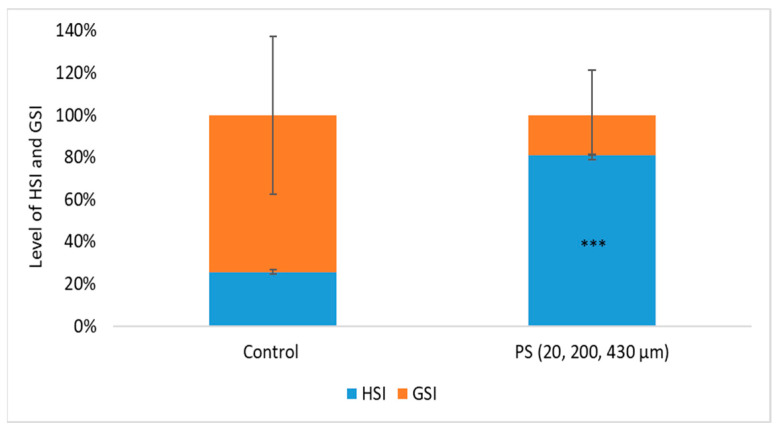
The level of hepato/gonadosomatic indexes (HSI/ GSI) after 75 days of exposure to PS mix (1.2 mg PS/L), *** *p* ≤ 0.001 indicates an extremely significant difference compared to control.

Biometric indices are very important in evaluating individual fitness and long-term changes in fish health and water quality. Based on these indicators, the following physiological indices were calculated: batch weight during the exposure period (W), feed consumption (C), growth coefficients (G), feed utilization efficiency for production (K%), production (P), biomass (B) (Table 1), and hepatosomatic (HSI) and gonadosomatic (IGS) indices (Figure 2).

From the results of physiological indices analysis (Table 2), the following aspects were highlighted: (i) The initial average individual weights (Wi) of the test and control groups do not differ significantly (*p* > 0.05). At the end of the exposure period, the final average individual weight (Wf) of the PS-treated group decreases slightly compared to the initial values but remain within the control limit. It should be mentioned that the control had an initial weight that was <10% lower than the tests group; (ii) the instantaneous growth coefficient (G) shows negative values for the PS-treated group, which correlates with the lower level of weight gain; (iii) the instantaneous mortality coefficient (Z) has not changed, it remains at the value of 0, as no mortalities were recorded during the tests; (iv) both initial and average biomasses (Bi and B) do not differ significantly (*p* > 0.05), remaining constant for both the control and PS-treated group; (v) production (P) is correlated with the instantaneous growth coefficient (G), indicating negative values; and (vi) the efficiency of use of food for production (K%) is correlated with both the instantaneous growth coefficient (G) and production (P), indicating a good food utilization for growth and development in the control, while showing less efficient use in the PS-treated group, although without major significance compared to the control groups. The findings align with existing literature highlighting that microplastics, especially nanoparticles, can inhibit growth performance by interfering with feeding behavior and nutrient absorption [60,61] and contribute to oxidative stress in fish [62]. By inducing a state of satiety or blocking digestive tracts, microplastics can reduce food intake efficiency, as observed in several fish species [63,64]. This may explain why feed utilization efficiency (K%) was lower in PS-exposed groups compared to controls.

### 3.6. Hepatosomatic and Gonadosomatic Index

During the chronic period exposure (75 days) to a PS mix of 20, 200, and 430 µm (1.2 mg PS /L, approx. 0.4 mg/L of each dimension), no mortality was recorded and no visible clinical signs were observed. In order to obtain information about the physiological health status of the internal organs, such as liver and gonads, which may be affected by long-term exposure to PS microplastics, the hepatosomatic index (HSI) and gonadosomatic index (GSI) were investigated. An increased HSI may indicate inflammatory responses due to toxic stress. A decreased GSI may suggest impaired reproductive function [65].

The HSI and GSI are shown in Figure 2. No significant differences in body mass between the control and exposed groups (*p* = 0.073) were observed, suggesting the homogeneity of the groups. Even so, the mean values of liver and gonad weights were examined, and the results show significant differences between the control group and PS-treated group based on hepatosomatic index (*p* = 0.00015). The PS exposure altered the GSI compared to the control group, although this change did not reach statistical significance (*p* = 0.164). The changes in HSI and GSI suggest that PS could influence processes initiated at the liver level, such as vitellogenesis, or at the ovarian follicle level, such as endocytosis of vitellogenin during the testing period. Studies on PS microplastics in fish revealed endocrine disruption effects in *Oryzias melastigma* [66], evidenced by GSI reduction in the 20 and 200 µg/L PS exposure groups, as well as damages to gonads resulting from of nano-PS accumulation in sex organs in *Danio rerio* [67]. This aligns with findings from our study regarding potential impacts on vitellogenesis and the gonad’s function.

### 3.7. Modulation of Enzyme-Specific Activities in Fish After Chronic Test

Our findings indicate tissue-specific toxicity in *C. carpio* exposed to the PS mix, revealing distinct responses in the intestine, kidney, liver, brain, gills, and gonads. The biochemical analyses revealed oxidative imbalances compared to the controls following chronic exposure of fish to the PS mix (Table 3).

**Figure 3:**
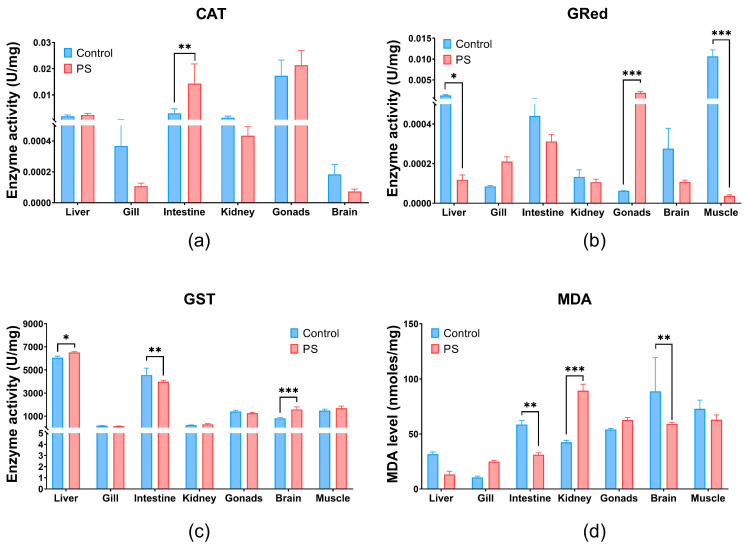
CAT (**a**), GRed (**b**), and GST (**c**) enzymatic activity, and MDA content (**d**) in liver, gill, intestine, kidney, gonads, brain, and muscle of *Cyprinus carpio* fish exposed to PS mix (1.2 mg PS/L) for 75 days. Data (n = 3 fish) are presented as means ± SD of enzymatic activity expressed in U/mg protein and for MDA in nmoles/mg protein. Statistical analysis was conducted using multiple *t*-tests (one per organ) and the Holm–Sidak method, with alpha = 0.05. * *p* ≤ 0.05, ** *p* ≤ 0.01; and *** *p* ≤ 0.001 control vs. PS-treated group.

Catalase (CAT) is an essential component of the adaptive response to hydrogen peroxide and is considered an essential biomarker for oxidative stress [68]. The PS mix used in our study caused a highly significant increase in CAT levels in the intestines (*p* ≤ 0.01) and, to a lesser extent, in the liver and gonads (no statistical significance). CAT enzymatic suppression in gills, kidneys, and brain compared to the controls after 75 days of exposure, although statistical significance was not reached (Figure 3a).

Glutathione reductase (GRed), shown in Figure 3b, is the enzyme that catalyzes the reduction reaction of oxidized glutathione, with the participation of NADPH [69]. Thus, it maintains the balance of reduced (GSH) and oxidized glutathione (GSSG) contents within cells. GRed activity increases under the influence of the PS mix in gonads (*p* ≤ 0.001) and in the gills without significance compared to controls. PS microplastics inhibits the GRed enzymatic activity in the liver, intestine, kidneys, brain, and muscle compared to the controls (Figure 3b). Significant inhibition of GRed activity were observed in muscle and gonads (*p* ≤ 0.001), but also in liver (*p* ≤ 0.05) (Figure 3b). Increased GRed activity in gonads could be an indicator of enhanced cellular mechanisms to combat oxidative stress and protect cells from oxidative damage.

Glutathione S-transferase (GST) plays an essential role in the detoxification process by removing xenobiotic compounds and providing protection against oxidative stress. GST is associated with a large number of compounds, acting as a regulatory protein. It catalyzes reactions involving GSH and an electrophilic substrate [70,71]. PS mix significantly increases GST enzymatic activity mainly in the brain (*p* ≤ 0.001) and liver (*p* ≤ 0.05) and with no statistical significance in kidneys and muscle. Conversely, GST levels significantly decrease in the intestines (*p* ≤ 0.01), but in gills and gonads no significance was observed. An elevation of GST activity can enhance the detoxification process by conjugating more toxic compounds with GSH, thus contributing to the overall antioxidant protection.

Lipid peroxidation (Figure 3d) measured indirectly by the formation of TBA-MDA adducts, showed a significant increase in the kidneys (*p* ≤ 0.001), and insignificant in gills and gonads. This finding is further supported by the enzymatic imbalances recorded at their level that trigger the antioxidant processes. The lipid peroxidation observed in the kidneys could be generated by contaminant stimulation through the production of reactive oxygen species (ROS).

Recent studies investigated the oxidative stress induced by microplastic on fish species such as *Carassius auratus* and *Larimichthys crocea*, highlighting various alteration in enzymes behavior, some of which confirm previously obtained results [72,73].

Regarding the oxidative stress, Usman (2021) reported that the intestine and brain was most affected by PS microplastics exposure [74]. The gills and gastrointestinal tract serve as entry points for microplastic accumulation. The antioxidant imbalance observed in the PS mix exposed intestine correlates with the accumulation of PS at this site, which was also confirmed through microscopic identification of PS particles in the intestinal contents of exposed fish and the CAT increased activity at this level.

The divergent results observed in enzymatic activities (CAT, GST, and GRed) and lipid peroxidation in fish exposed to PS microplastics can be attributed to compensatory antioxidant mechanisms [75]. Alongside GST and GRed, other enzymes such as CAT, superoxide dismutase (SOD), and glutathione peroxidase (GPx) play critical roles in defending against oxidative stress. Although SOD and GPx were not assessed in this study, these enzymes may compensate for reduced GST and Gred activities, thereby maintaining lipid peroxidation at a low level. This could explain the apparently contradictory results. Furthermore, interdependent enzymatic regulation systems can be activated [76], as evidenced in our study, by significantly increased GST activity and decreased GRed activity in the liver.

This interaction suggests that compensatory mechanisms could mask lipid peroxidation effects. Prolonged exposure to oxidative stress, over 75 days, may lead to a cellular adaptation, resulting in reduced GRed expression in the liver, intestine, kidneys, brain, and muscles. Additionally, external stress factors, such as captivity, cannot be excluded [77].

The PS mix caused changes in the activity of CAT and GRed enzymes correlated with lipid peroxidation in the gills, but without significant effects suggesting a good defense system at this level. In the liver, an organ responsible for the detoxification, the antioxidant enzyme revealed moderate effects without lipid peroxidation reactions. This observation suggests an adaptive response to the PS mix and efficient counteraction of oxidative events through defense mechanisms.

A study on PS microplastics revealed that the small particles accumulate in the respiratory and digestive systems of fish, and thus, biochemical changes can occur up to lipid peroxidation. PS microplastics can induce oxidative damage to lipids and the production of ROS, which finally results in tissue injury [78].

The decreased CAT activity observed—responsible for degrading hydrogen peroxide accumulated after PS exposure—indicates an inhibition of the normal ROS elimination process. Wang X. et al. (2022) demonstrated that exposure to 100 or 1000 μg/L PS particles (size of 0.5 µm or 5 µm) decreased CAT activity due to excessive H_2_O_2_ accumulated in the organ, which may result in tissues damage [60].

The liver injury observed in *C. carpio*, in our study, indicated by changes in antioxidant enzyme levels, is supported by Cui et al. (2023) [79], who reported abnormal liver function and inflammatory effects induced by ROS accumulation. Similarly, Banaei et al. (2022) noted the inhibition of enzymatic activity (CAT and GRed) in the liver and intestine following exposure to polyethylene microplastics at concentrations of 350–1400 µg/L for 30 days. These effects were attributed to ROS overproduction or alterations in gene expression. Variations in antioxidant enzyme responses may reflect changes in intracellular ROS concentrations [80].

### 3.8. EROD, VTG, and ACh Enzyme Specific Activities

**Figure 4:**
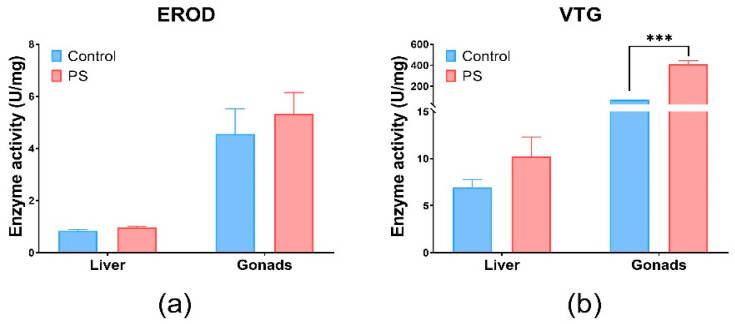
EROD (**a**) and VTG (**b**) enzymatic activity in fish liver and gonads. *Cyprinus carpio* fish was exposed to PS mix (1.2 mg PS/L) for 75 days. Data (n = 3 individuals) are presented as means ± SD of enzymatic activity expressed in U/mg protein. Statistical analysis was conducted using multiple *t*-tests (one per organ) and the Holm–Sidak method, with alpha = 0.05. *** *p* ≤ 0.001 control vs. PS-exposed group.

The EROD activity was indirectly investigated through the enzyme Cytochrome P450 monooxygenase 1A (CYP1A1) a biomarker for xenobiotics, using the ELISA technique with specific antibodies [81]. The activity of the EROD enzyme indicating the presence of CYP1A1 detoxification enzymes, without statistical significance (*p* > 0.05) in the liver and gonads, correlated with antioxidant enzyme and lipid peroxidation in these organs (Figure 4a). The insignificant expression of EROD activity (*p* > 0.05) compared to the control group, in our experiments, suggests induction by ROS metabolism. Another study confirms that the microplastics did not affect its activity [82].

Vitellogenin (VTG), which is involved in the reproduction process, was measured by the ELISA technique with specific antibodies to carp vitellogenin (Figure 4b). Changes in VTG levels can be correlated with gonadosomatic and hepatosomatic indices, which showed alterations compared to controls. Significant effects were observed in the gonads (*p* < 0.001). The possibility of an estrogenic impact of PS is not excluded, as it correlates with the inhibition of several enzymes involved in antioxidant defense, such as CAT and GRed at the gonads level, as well as a slight increase in lipid peroxidation levels. Moreover, according to literature, PS microplastics could exhibit estrogenic effects in fish [83]. The changes in the VTG and GSI levels observed after PS contamination suggest potential estrogenic effects. Fish vitellogenin is a common estrogenic biomarker of aquatic pollutants [84]. The literature revealed that microplastics could downregulate the transcription of vitellogenin in the liver of fish [85].

**Figure 5:**
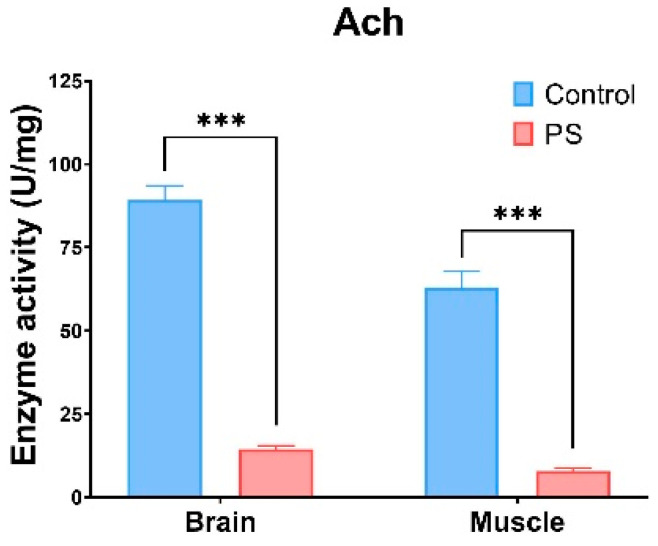
ACh enzymatic activity in fish brain and muscle. *Cyprinus carpio* fish was exposed to the PS mix (1.2 mg PS/L) for 75 days. Data (n = 3 individuals) are presented as means ± SD of enzymatic activity expressed in U/mg protein. Statistical analysis was conducted using multiple *t*-tests (one per organ) and the Holm–Sidak method, with alpha = 0.05. *** *p* ≤ 0.001 control vs. PS-treated group.

ACh refers to enzymes that hydrolyze the neurotransmitter acetylcholine into acetate and choline. Changes in ACh activity can result from exposure to certain chemical substances, which act as cholinesterase inhibitors. ACh, which is involved in the neurotransmitter processes in the brain and muscles, is inhibited by the presence of microplastics [86,87,88]. It can be observed that the PS mix induces a similar type of response in both organs analyzed (brain and muscle), with a recorded a reduction of 80–90% in ACh activity compared to the control group (*p* ≤ 0.001) (Figure 5).

According to the literature, microplastics can exert size-dependent toxicity [78]. Some studies have shown that microplastics smaller than 10 µm can cross intestinal tissues and enter the bloodstream, potentially affecting the brain [89,90]. In our study, the size of PS particles was in the range of 20, 200, and 430 µm, which complicates the explanation for observed brain effects due to their larger size. However, if the PS particle suspensions included undetected particles smaller than 20 µm, this could account for neurological impact. Previous research highlighted ACh inhibition when exposed to microplastics of micrometer size [91]. Notably, hypoactivity was reported only with 10 µm PS microplastics in zebrafish larvae [92].

Our results demonstrate that exposure to the PS mix led to significant changes in ACh activities in *C. carpio*. ACh is crucial for cholinergic neurotransmission at neuromuscular junctions and cholinergic synapses in the brain [93]. The observed decrease in ACh activity in both brain and muscle indicates that PS could significantly disrupt cell signaling pathways (*p* < 0.001). This reduction in ACh activity may also result from oxidative damage within these tissues. Similar decreases in ACh activity have been documented in *Symphysodon aequifasciatus* [94] and *Pomatoschistus microps* [95] exposed to microplastics.

The activities of CAT, GRed, and GST in the brains of fish intoxicated with the PS mix correlate with the reduction in ACh activity, which is also confirmed at the muscle level. Interestingly, no visible clinical signs were observed at the individual level. This lack of observable symptoms can be attributed to either compensatory stress adaptation mechanisms or the possibility that the tested dose (1.2 mg/L) was too low or that the exposure time (75 days) was insufficient.

Another recent study on fish suggests that oxidative stress in the intestine and brain, as well as tissue alterations in the kidneys and liver, highlight the importance of the gut–brain axis in understanding physiological interactions related to microplastic contamination [74].

### 3.9. Hepatic Enzyme Specific Activities

**Figure 6:**
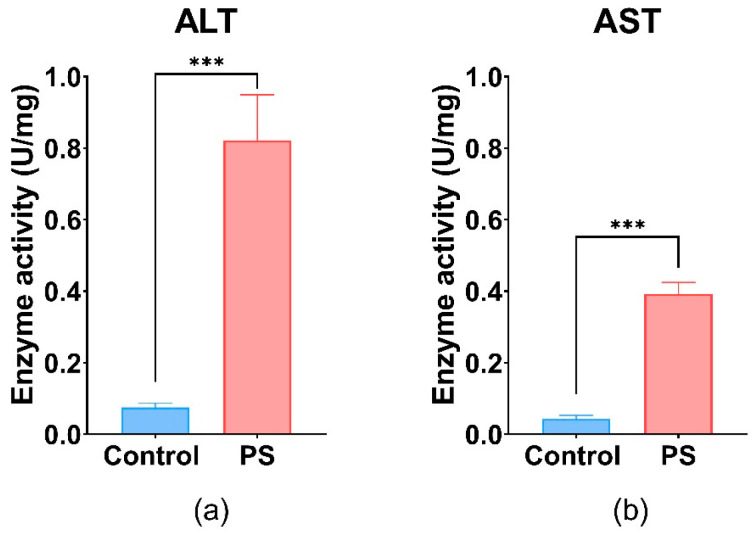
ALT (**a**), and AST (**b**) enzymatic activity in fish liver exposed to PS mix (1.2 mg PS/L) for 75 days. Data (n = 3 individuals) are presented as means ± SD of enzymatic activity expressed in U/mg protein. Statistical analysis was conducted using a two-tailed student *t*-test. *** *p* ≤ 0.001 indicates an extremely significant difference vs. PS-treated group.

Liver activity measured by AST and ALT shows important differences between the control and PS-treated group. The values obtained for both enzymes were statistically significant, suggesting liver injury (Figure 6). PS contamination could have an impact on the liver and gonads regarding the physiological processes such as detoxification, vitelogenesis, and endocytosis.

These enzymes are involved in processes such as glutathione biosynthesis, gluconeogenesis in liver hepatocytes, and the maintenance of NAD+/NADH equilibrium, as well as neurotransmitter synthesis [96]. Furthermore, the increase in hepatic enzyme activities (AST and ALT) in the liver (*p* ≤ 0.001) after 75 days of exposure could indicate oxidative damage, pointing to the presence of oxidative stress and potential tissue damage in *C. carpio* following PS exposure. Literature reported that exposure to various types of microplastics, including polystyrene, has been shown to significantly increase hepatic enzyme activities such as AST and ALT in *Cyprinus carpio*, indicating liver injury associated with oxidative stress and inflammation [97].

### 3.10. Protein Expression Profile

**Figure 7:**
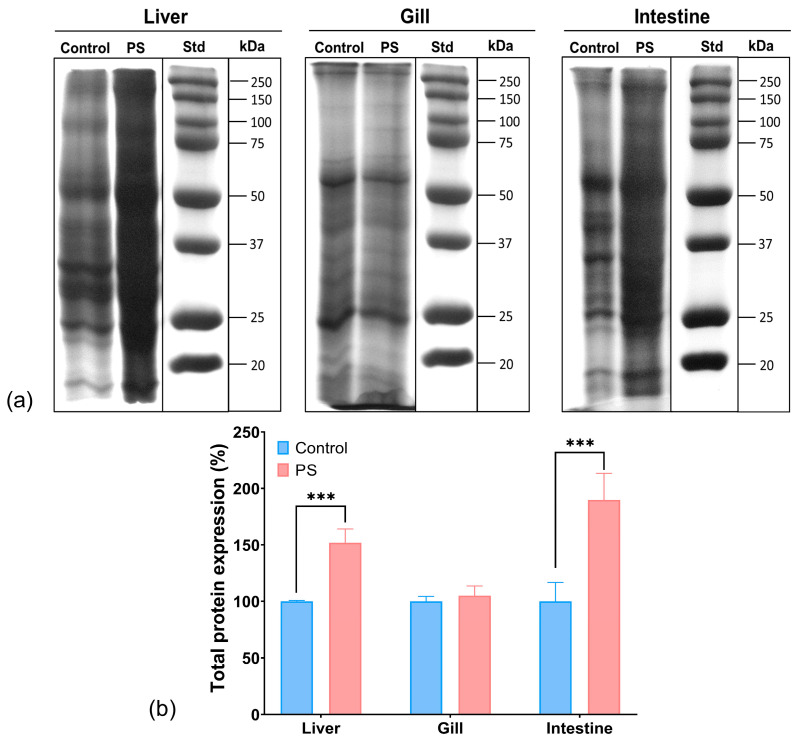
Protein expression profile obtained by SDS-PAGE electrophoresis in the liver, gill, and intestine of unexposed fish and exposed for 75 days to PS mix at a concentration of 1.2 mg PS/L. Electrophoresis gels (**a**); the graph (**b**) illustrates the quantification of total protein expression (n = 3 individuals) expressed in percentage. Statistical analysis was conducted using multiple *t*-tests (one per organ) and the Holm–Sidak method, with alpha = 0.05. *** *p* ≤ 0.001 indicates an extremely significant difference vs. PS-treated group.

The SDS-PAGE analysis of the protein extracts from the organs of *C. carpio* groups, control and treated with PS mix for 75 days, revealed a different protein profile in the liver and intestine of the PS-treated group compared to control. No differences were observed between the groups regarding the protein expression profile in the gills. It was noted that the total protein expression in the liver increased by 52%, while in the intestine, it increased by 90% compared to the control (Figure 7). The enhancement of protein expression may indicate transcriptional activation for the synthesis of various proteins, possibly as a result of oxidative stress.

Moreover, the smeared appearance of protein bands in the liver and intestine samples might also indicate proteolytic or oxidative degradation processes, potentially generating smaller peptide fragments or oxidized proteins, as well as the formation of protein aggregations and complexes in fish organs in the presence of microplastic pollutants. While these data do not provide details on specific stress-related proteins, they suggest a possible harmful effect of PS that may result in an amplification of protein biosynthesis and degradation. Similarly, scientific literature reported changes in the expression of stress proteins following PS contamination in fish [80].

The changes in protein expression observed in this study may be associated with inflammatory responses triggered by microplastic exposure. Research indicates that microplastics can activate immune-related genes involved in inflammatory signalling, potentially leading to alterations in protein expression as part of a broader stress response [64].

The significant increase in intestinal protein expression aligns with findings from other studies, which have shown that microplastics can cause structural damage to intestinal tissues, such as shortening of villi and depletion of goblet cells. These changes may require enhanced protein synthesis for tissue repair [98].

## 4. Conclusions

This study investigated the acute and chronic toxicity of polystyrene (PS) microparticles on *Cyprinus carpio* (common carp), highlighting their effects on oxidative stress, liver metabolism, and enzymatic systems involved in reproduction and neurotoxicity.

The results show that while PS exposure in fish did not cause mortality or observable clinical signs (up to 100 mg/L), significant biochemical alterations were detected when the fish were exposed to a mix of PS in different particle sizes. Oxidative stress biomarkers (CAT, GRed, GST, and EROD) were disrupted, particularly in the intestine, liver, and gonads. Lipid peroxidation levels were notably increased in the kidneys, gills, and gonads, indicating oxidative damage and potential cellular stress. Elevated ALT and AST enzyme levels were detected, suggesting potential liver damage, while the inhibition of acetylcholinesterase activity in the brain and muscle indicated possible neurotoxic effects. Additionally, the increase in VTG, along with modifications in hepatosomatic and gonadosomatic indices, indicates a systemic response to PS exposure.

The study also revealed a significant increase in total protein expression in the liver and intestines, suggesting an adaptive response to oxidative stress. Although these biochemical changes did not translate into immediate lethal effects, they indicate potential long-term physiological consequences. Moreover, the presence of microplastics in commercially important fish species poses potential risks to human health. As these fish are consumed by humans, there is a growing concern about the transfer of microplastics and associated toxins into the human food chain.

The findings emphasize the importance of monitoring microplastic contamination in aquatic environments, as prolonged exposure to PS microparticles may disrupt oxidative homeostasis in fish and other aquatic organisms.

Further studies are still needed to elucidate the long-term exposure of fish to different types of microplastics from different sources (especially textile, tires, and construction material), in various sizes and mixt that closely mimic aquatic environmental conditions.

Ecotoxicological studies of microplastic interactions with other types of pollutants, such as pesticide, pharmaceutical compounds, metals, and bacteria, need to be conducted. Additionally, endocrine and neurotoxic effects should be investigated more thoroughly. Due to the specificity of these pollutants, detection, characterization, and ecotoxicity assessment standards are considered necessary.

## Figures and Tables

**Table 1:** Weight, length, and height of specimens at 0 h and 75 days in the chronic test.

Parameter	Biometric Data			
	Control		PS	
	T (0 Days)	T (75 Days)	T (0 Days)	T (75 Days)
	Weight (g)			
Total weight of fish batch (10 individuals)	677	686	622	586
Average of weight (n = 10)	67.70	68.60	62.20	58.60
SD	15.29	15.00	14.92	14.02
CV %	22.00	23.00	23.99	23.92
Food (1% of the weight of the batch)—grams	6.77	-	6.22	-
	Total length (cm)			
Average of length (n = 10)	15.95	15.62	15	15.67
SD	1.46	1.86	1.13	1.28
CV %	10.47	12.77	7.53	8.18
	Height (cm)			
Average of height (n = 10)	4.65	4.72	4.64	4.6
SD	0.42	0.33	0.34	0.39
CV %	9.99	7.77	7.41	8.57

Notes: SD—standard deviation; CV—variation coefficient (%); and PS—polystyrene mix of particles of size 20, 200, and 430 µm.

**Table 2:** Values of the physiological indices after 75 days of exposure to PS.

Test	Time (Days)	Ni	Wi (g)	Nf	Wf (g)	G	Z	Bi (g)	B (g)	P(g)	C (g)	K%
Control	75	10	67.70	10	68.60	0.0002	0.00	677.00	677.06	0.12	372.35	0.03
PS mix	75	10	62.20	10	58.60	−0.0008	0.00	622.00	621.75	−0.49	342.10	−0.14

Notes: PS mix—polystyrene mix in particle size of 20, 200, 430 µm; Ni—initial number of specimens; Wi—initial average individual weights (g); Nf—final number of specimens; Wf—initial average individual weight (g); G—instantaneous growth coefficient; Z—instantaneous mortality coefficient; K—feed utilization efficiency for production (%); Bi—initial biomass (g); B—biomass average (g); P—production (g); and C—feed consumption (g).

**Table 3:** Significance of enzymatic activity changes in oxidative stress enzymes in fish organs after chronic exposure to a PS mix.

Organ	CAT	GRed	GST	MDA
Liver	− (ns)	− (*)	− (*)	− (ns)
Gills	− (ns)	+ (ns)	− (ns)	+ (ns)
Intestine	+ (**)	− (ns)	− (**)	− (**)
Kidneys	− (ns)	− (ns)	+ (ns)	+ (***)
Gonads	− (ns)	+ (***)	− (ns)	+ (ns)
Brain	− (ns)	− (ns)	+ (***)	− (**)
Muscle	NA	− (***)	+ (ns)	− (ns)

Notes: symbol “−” inhibition of enzymatic activity; symbol “+” increase in enzymatic activity. *p* > 0.05 indicates a no statistically significant difference (ns); * *p* ≤ 0.05 indicates a statistically significant difference; ** *p* ≤ 0.01 indicates a highly significant difference, and *** *p* ≤ 0.001 indicates an extremely significant difference compared to control. NA—undetermined.

## Data Availability

Data is contained within the article.

## Supplementary Materials

The following supporting information can be downloaded at: https://www.mdpi.com/article/10.3390/toxics13040246/s1, Table S1. The physical-chemical parameters monitored in the acute toxicity test with PS mix (average of *n* = 4 ± SD); Table S2. Monitoring of the physical and chemical parameters necessary for survival during the chronic exposure period.
