# Supplementary material for: Ecotoxicological Effects of Polystyrene Particle Mix (20, 200, and 430 µm) on Cyprinus carpio
Source: Toxics. 2025 Mar 26;13(4):246. doi: 10.3390/toxics13040246 (PMC12031100; doi:10.3390/toxics13040246)
Supplement: Supplementary file 1 [file toxics-13-00246-s001.zip › toxics-3504214-supplementary.pdf]

Supplementary Materials

Ecotoxicological Effects of Polystyrene Particle Mix (20, 200, and 430 μm) on *Cyprinus carpio*

Ştefania Gheorghe [1,*,†], Anca-Maria Pătraşcu [1,2,†], Cătălina Stoica [1,†], Mihaela Balaş [3,*] and Laura Feodorov [1,4]

[1] Control Pollution Department, National Research and Development Institute for Industrial Ecology ECOIND, 57-73, Drumul Podu Dambovitei Str., 060652 Bucharest, Romania
[2] Faculty of Biotechnical Systems Engineering, National University of Science and Technology Polyethnic, 313 Splaiul Independentei, 060042 Bucharest, Romania
[3] Department of Biochemistry and Molecular Biology, Faculty of Biology, University of Bucharest, 91-95 Splaiul Independentei, 050095 Bucharest, Romania
[4] Faculty of Biotechnology, University of Agronomic Sciences and Veterinary Medicine of Bucharest, 59 Marasti Blvd, District 1, 011464 Bucharest, Romania
* Correspondence: stefania.gheorghe@incdecoind.ro (Ş.G.); mihaela.balas@bio.unibuc.ro (M.B.)
† These authors contributed equally to this work.

Table S1. The physical-chemical parameters monitored in the acute toxicity test with PS mix (average of *n*=4 ± SD)

Parameter	Initial (0h)	Final (96h)
**Control (dilution water)**		
Dissolved oxygen concentration (mgO2 /L)	6.30±0.54	6.37±0.58
pH (pH units)	7.64±1.20	7.76±1.04
Conductivity (μS/cm)	220.60±20.30	354.10±26.60
Temperature (°C)	21.20±0.50	20.80±0.50
COD (mgO2 /L)	35.21±13.10	64.40±24.41
**Test solution (PS mix)**		
Dissolved oxygen concentration (mgO 2 /l)	6.55±1.63	6.20±1.30
pH (pH units)	7.35±0.52	7.75±1.01
Conductivity (μS/cm)	260.90±20.53	362.50±23.50
Temperature (°C)	21.40±0.50	20.40±0.50
COD (mgO2 /L)	41.00±15.00	69.80±38.35

Table S2. Monitoring of the physical and chemical parameters necessary for survival during the chronic exposure period

	pH (pH units)		Conductivity (μS/cm)		Dissolved oxygen (mgO2/L)		Temperature (°C)	
	Control	PS	Control	PS	Control	PS	Control	PS
Average (n=45)	7.69	7.58	275.75	260.80	7.27	7.04	22.10	22.30
SD	0.24	0.16	4.47	32.92	0.27	0.51	0.98	0.80
CV%	3.19	2.15	1.62	12.62	3.83	4.79	4.43	3.58

Note: SD – standard deviation; CV – variation coefficient (%); PS – polystyrene mix of particles of size 20, 200, 430 μm
